# Prof. J. P. Dake, M. D.,—Personal Recollections

**Published:** 1894-12

**Authors:** Bushrod W. James

**Affiliations:** Philadelphia, Pa.


### PROFESSOR JABEZ P. DAKE, A. M., M. D.—PERSONAL RECOLLECTIONS.

Bushrod W. James, M. D., Philadelphia, Pa.

(Read before the Memorial Meeting held at Philadelphia, Nov. 15th, 1894.)

The death of my intimate friend, Dr. Jabez P. Dake, brings to my recollection our pleasant relations from the days in which he was our Professor of Materia Medica, in the Homœopathic College of Pennsylvania, in my native city in 1856 and 1857, until his death.

He was not a stern, pedantic instructor, whose erudition lifted him far above the level of those whom he taught. He was kind, considerate, and helpful, bearing patiently with those who were slow to learn, and appreciating those who were gifted beyond their fellows, yet withal, conscientiously careful to display no favoritism.

His sincerity gained respect, his urbanity and dignity held my life-long regard, while there was an attraction in his cultivated voice and a winning warmth in his smile that won my affection from our first acquaintance.

Dr. Dake became a member of the American Institute of Homœopathy in 1852, and from that time his strong and active interest in its welfare never flagged, even in the last days of his life. He became an active member at once, and never grew weary of striving for the furtherance of every advanced idea in which he discovered intrinsic worth, but he always strenuously opposed that which was contrary to the true, forceful law of Homœopathy. Because of this the Doctor met with considerable antagonism at times, but in facing it he was always courteous, never forgetting the ethical laws which were deeply instilled and so thoroughly taught in our colleges, and by our preceptors and professors, in those days.

In 1857, Dr. Dake was elected President of the American Institute of Homœopathy, and in 1859 I became a member, and read with deep interest the annual address delivered by him in Brooklyn, in 1858, at the American Institute's annual meeting. Scholarly, instructive, and directed with firm conviction, it gave promise of what the young physician would become in his future professional life. Soon after we became firm friends, oftentimes thrown together in debates and in the many forms of companionship arising from our co-equal interest in the progress of the science of Homœopathy, and its final almost universal adoption for the cure of suffering humanity.

In 1878 Dr. Dake served on the Yellow Fever Commission which visited New Orleans, Memphis, and other places in the South in which the scourge was severely felt, and in that serious time one was led to admire his noble faithfulness and tireless unselfishness. He simply forgot himself in the misery of others, and in his zeal to prove the efficacy of the new mode of treatment. He lost sight of the possibility of his health succumbing to the climate, the disease-laden atmosphere, or his indefatigable devotion to his profession. There were some enthusiastic men who formed that commission, among whom conspicuously stood out the President, Dr. William H. Holcombe, of New Orleans, who had experience from several visitations of the plague in his own city, and was an eminently successful practitioner.

Dr. Dake was a prominent, active, and just member of the "Senate of Seniors," an association attached to the American Institute of Homœopathy, and consisting of members who have been connected with the Institute for twenty-five years. To them peculiar questions are committed for arbitration, and no member was ever more consistently disinterested or wisely faithful to the trust than our honest friend. His sincere devotion to the Institute and the promotion of its welfare could not be more clearly demonstrated than in a letter which I received a day or two before he died, in which he speaks most earnestly and trustingly of its continued usefulness.

In his own home the Doctor was graciously hospitable, but I think in no other relation was he more truly admirable than as a traveling companion. I can safely aver that some of the happiest days I ever knew were spent in his company. As we journeyed through England, Russia, Sweden, Norway, Finland, Denmark, and other countries his glowing appreciation of every new scene, his love for nature in mountain, valley, and river added new delight to the enjoyment already stirred in my own heart by their ever-changing beauty. Vivid pictures are specially recalled to me, now that I am made to mourn the absence forever of the companion by whose side I enjoyed their loveliness.

In Copenhagen, Stockholm, Christiana, Abo, and other cities we visited churches, castles, and public institutions, my friend always discovering the rarest beauties in architecture and paintings, always pausing to drink in the very soul of the artist's ideas, whether in the one or the other. At Copenhagen, in company with others, we made a visit to an institution in which a Masseur was demonstrating the Swedish movements. He invited us in and gave us a fine exhibition of his skill in the art of massage upon a patient.

While visiting Norway we made an excursion from Christiana to several spots of historic interest in the neighborhood, among them being Krongkleven, meaning a rocky cliff, about 1,300 feet above the level of the city. After stopping to look at each change in the wonderful panorama on our way, we rode on to a spot called Kongen Udsigt, or King's View. There we sat upon a rock, hewn by Nature into the resemblance of a rude chair, and gazed in speechless delight at the glorious view which opened upon our vision. Away to the northwest we saw the Tyrifjord, its many islands resting like gems in the sparkling water; beautiful, quaint little towns and hamlets, with arms of the Tyrifjord glinting here and there through verdant valleys, and forests of bright green trees waving in the gentle wind. In the west we saw in the distance Gausta and other snow-clad mountains, making a strong, cool contrast with the lovely beauty of the country at their feet.

Only one who has been thus comparatively alone with Nature in her untrammelled beauty, with neither art nor science to improve or mar, can realize how closely one is drawn to an enthusiastic companion, whose enjoyment, too full for mere words, finds expression in soulful breath and sparkling eyes. So Dr. Dake and I sat side by side, gazing in wondering silence at the exquisite loveliness of those islands, waters, valleys, and mountains, and I doubt not, he as well as I, thought of how glorious the land must be that is more beautiful than this fair earth. And now he beholds that other country, that perfect land, while my heart swells with grief for the loss of my dear friend, in whose companionship I took such pleasure.

I can recall most clearly another scene in which we found food for many a thought. We took the steamer from Stockholm to Abo, in Finland, and then to St. Petersburg, and never will I forget that beautiful sail! It was about four o'clock in the afternoon when we started, and the voyage extended into the next day. But we were near the Land of the Midnight Sun, and no darkness came to shut out the view as we sailed around and among a myriad of verdant islands. Some were but tiny knolls above the shimmering waters, others were merely rocky spaces for the most part, but larger, and covered with bright green grass and shrubbery. As we steamed through that lovely highway in the Baltic, now from island to island, then for hours through the broad sweep of waters with no land in sight, and again within touch of emerald islands, before we reached Finland's rocky coast, the sun set and rose again, leaving the earth so small a space of time that even midnight was not dark. The grand old orb seemed to make a long, graceful sweep from west to east, the light from his wake meeting the promise of the new morning in a soft twilight which touched water, sky, and island with the most tenderly beautiful shadow. And then the morning burst forth, in rose and pink and gold, exquisite in its greeting to the smiling earth.

We were companions, brothers then, heart answering heart in responsive joy at the splendor of the world and the majesty of that globe of light which glorified all things in its generous beauty.

But now he has gone beyond! And I cannot yet see the more glorious day that has risen to greet him.

Always now I will feel lonely when those past days come before me. I will miss him always—my teacher, friend, companion, whose heart was true, and whose friendship and love were an honor upon whomsoever they were bestowed.

And now a change has come, and he has found
All beauty perfected, that in this sphere
In buds of joyous promise blessed the ground,
Or broke in sweet, low music on his ear.
No darkness dims the beauty to his sight,
Nor sorrow touches, with its pain, his heart.
Uplifted, he beholds in glorious light
Scenes that makes thoughts of earthly things depart.
We mourn, but he feels not our lonely pain,
Nor knows how sad the space his flight has left.
Nor would we ask to call him hence again
Though grief lies heavy in our hearts bereft.
And now we say "good-bye," and rest in peace,
Dear honored friend, companion tried and true—
Safe where the cares of life forever cease—
We bid thee for awhile a fond adieu!

